# Economic Aspects in the Management of Diabetic Macular Edema in Italy

**DOI:** 10.3389/fpubh.2022.938987

**Published:** 2022-07-22

**Authors:** Giovanna Elisa Calabrò, Michele Basile, Monica Varano, Filippo Amore, Roberto Ricciardi, Francesco Bandello, Americo Cicchetti

**Affiliations:** ^1^Section of Hygiene, University Department of Life Sciences and Public Health, Università Cattolica del Sacro Cuore, Rome, Italy; ^2^VIHTALI (Value in Health Technology and Academy for Leadership and Innovation), Spin-Off of Università Cattolica del Sacro Cuore, Rome, Italy; ^3^Graduate School of Health Economics and Management (ALTEMS), Università Cattolica del Sacro Cuore, Rome, Italy; ^4^Fondazione Bietti-IRCCS, Rome, Italy; ^5^IAPB Italia Onlus - Polo Nazionale di Servizi e Ricerca per la Prevenzione della Cecità e la Riabilitazione Visiva, Centro di Collaborazione Organizzazione Mondiale Sanità Presso Fondazione Policlinico Universitario “A. Gemelli” IRCCS, Rome, Italy; ^6^Department of Ophthalmology, Scientific Institute San Raffaele, University Vita-Salute, Milan, Italy

**Keywords:** diabetic macular edema, economic burden, intravitreal dexamethasone implant, direct costs, indirect costs

## Abstract

**Background:**

Diabetic Macular Edema (DME) is the most common cause of vision loss in diabetic patients. Currently, the Vascular Endothelial Growth Factor inhibitors (anti-VEGFs) are used as the first line of DME treatment and corticosteroid implants are usually used as a second-line treatment. These implants are a safe and effective therapeutic option that can improve the quality of life of DME patients by reducing the intravitreal injections number. We determined the economic impact related to DME, also from the social perspective, and the consequences of the increased use of the dexamethasone implant.

**Methods:**

The analysis compares two scenarios: the first based on the current rate of recourse to the therapeutic alternatives available in the Italian healthcare setting (as is) and the second based on the assumption of an increased recourse to dexamethasone implants (to be). The results are expressed both in terms of the resource absorption associated with the two scenarios and in terms of the cost differential yielded by their comparison.

**Results:**

The increased use of the dexamethasone implant allows considerable savings in terms of healthcare professionals' time, follow-up and productivity lost by patients/caregivers. These savings would reduce healthcare costs for the management of DME patients in Italy by €2,058,238 in 5 years.

**Conclusions:**

To optimize the healthcare resources allocation, it is necessary to implement treatments that yield not only cost reductions but also a clinical benefit for patients. The dexamethasone implant use is an example of DME management that generates value for patients, health system and society.

## Introduction

Diabetic macular edema (DME) is the main cause of vision loss in patients with diabetic retinopathy (DR) which is a major complication of diabetes (1).

Today, diabetes mellitus (DM) is a major health emergency. The World Health Organization (WHO) estimates that around 422 million people worldwide suffer from diabetes and 1.5 million deaths are directly attributed to the disease each year (2). Its global prevalence has nearly doubled since 1980, rising from 4.7 to 8.5% in the adult population, and this datum reflects an increase in associated risk factors, such as overweight and obesity (3). According to 2021 data from the International Diabetes Federation (IDF), the prevalence of diabetes in Europe is 9.2%, 1 in 11 adults have diabetes (61 million) and 1 in 3 (33%) diabetic subjects are undiagnosed. The disease prevalence will see a 13% increase by 2045 (4). In Italy, the overall prevalence of diabetes is 6.2%; two-thirds of diabetic subjects (66.9%) are aged ≥ 65 years, almost one-third are of working age (20–64 years) and 23% are aged ≥ 80 years (5).

Most diabetics have type 2 diabetes, which accounts for over 95% of cases worldwide (2). If diabetes is not adequately managed, patients face major complications, both acute and chronic (e.g., infections, myocardial infarction, stroke, amputations, end-stage renal disease and blindness), which may be life-threatening (5). These complications contribute substantially to mortality, poor quality of life, personal and social medical costs and overall healthcare expenditure in most countries (3, 5).

DR is a common complication of DM and a leading preventable cause of blindness in the adult working population (6). According to the Global Burden of Disease Study, DR is the fifth main cause of blindness and of moderate/severe vision impairment in adults aged 50 years and older (7). Among diabetic patients, the global prevalence rates of DR, vision-threatening DR (VTDR) and clinically significant macular edema (CSME) are estimated to be 22.27, 6.17, and 4.07%, respectively, (6). In 2020, the numbers of adults worldwide with DR, VTDR, and CSME were estimated at 103.12, 28.54, and 18.83 million, respectively, and by 2045, these numbers are projected to increase to 160.50, 44.82, and 28.61 million, respectively, (6). As the population ages and risk factors for DM related to improper lifestyles increase, a greater burden of DR and DME and an increased demand for eye care are expected in the coming years (8).

DME is the main cause of moderate vision loss among diabetic subjects worldwide, and for several years grid and focal laser photocoagulation were considered the standard of care for this eye disease (9). However, according to the European Society of Retina Specialists (EURETINA) guidelines, focal/grid laser therapy is now reserved mostly for non-center-involving DME (10). In recent years, the introduction into clinical practice of intravitreal therapy with vascular endothelial growth factor inhibitors (anti-VEGFs) and corticosteroid implants has revolutionized the medical management of DME (11). Indeed, these treatments result in a significant improvement in the best-corrected visual acuity (BCVA) at 12 months in patients with DME (11).

However, despite the clinical benefits of anti-VEGFs, these agents (in Italy, bevacizumab, ranibizumab and aflibercept are currently authorized) require several intravitreal injections and follow-up visits; this means not only high costs for the health system (12) but also significant indirect costs for patients, their families and society (13). Moreover, as patient compliance with these therapeutic regimens is suboptimal, the clinical efficacy of these treatments following their suspension is low (14).

Currently, anti-VEGFs are used as the first line of treatment for DME; however, many patients do not respond adequately to them, and corticosteroid implants are usually used as a second-line treatment (15). In 2014, an intravitreal implant with 700 μg of sustained-release biodegradable dexamethasone (Ozurdex®, Allergan, Inc., Irvine, CA, United States) was approved by the Food and Drug Administration (FDA) for DME treatment. The MEAD study demonstrated the efficacy of the dexamethasone implant, reporting an acceptable safety profile and a low number of injections (four or five over a 3-year follow-up) (16). Its efficacy following a single injection extends beyond 4 months, achieving good stabilization until the 12th month, with 2.13 injections during this period; this confirms the good anatomical and functional effectiveness in treating DME in real-life clinical practice (17).

The use of long-acting drugs can improve the quality of life of patients by reducing the frequency of intravitreal injections; moreover, the need for frequent injections may lead to a reduction in compliance with therapy, thereby reducing treatment efficacy (14). Furthermore, the use of these therapies could also result in cost savings for the health system and for society (14). Therefore, the purpose of this study was to estimate the economic burden of DME in Italy, to analyze the economic consequences associated with a greater use of the dexamethasone implant in the Italian setting, and thus support policy-makers in designing value-based strategies directed to tackling this growing disease.

## Materials and Methods

We assessed the economic burden of DME in Italy by considering both direct healthcare costs and productivity losses. Specifically, the analysis involved a comparison between two alternative scenarios:

- Scenario 1 (*as is*), based on current clinical practice (anti-VEGFs as the first line of DME treatment and the sustained-release dexamethasone implant as the second line).
- Scenario 2 (*to be*), based on the assumption of an increased recourse to the sustained-release dexamethasone implant in the Italian healthcare context.

### Direct Costs

A Budget Impact Analysis (BIA) was made from the perspective of the National Health Service (NHS), in order to estimate the financial impact associated with the increased use of the dexamethasone implant, as compared with the anti-VEGF alternatives currently available in Italy (ranibizumab, aflibercept and bevacizumab).

The BIA included the following input data:

1. Population eligible for DME treatment (target population);
2. DME epidemiology in Italy;
3. DME treatment costs;
4. Costs of DME management and follow-up.

The analysis considered a 5-year time horizon. The input data of the economic model were validated by two clinicians with proven experience in the management of DME patients. In order to assess the robustness of the results obtained in the analysis, a one-way sensitivity analysis (OWA) was carried out to determine the drivers whose variation most affected the estimates made in the base-case scenario. Each parameter included in the OWA was associated with a level of uncertainty of 25% of its average value. The results are shown in terms of the difference in resource consumption between the two alternative scenarios considered in the analysis.

#### Target Population and Epidemiology of DME in Italy

The target population was calculated on the basis of the Italian population in 2020 and consisted of 59,236,213 individuals (18). This population was weighted by the epidemiological indexes of prevalence characterizing the disease, in order to estimate the number of DME patients in Italy. Specifically, a 5.89% prevalence rate of diabetes (2, 5), obtained by weighting the prevalence of diabetes in Italy (6,2%) (5) by the share of patients developing type II (95%) (2), and a 6.81% prevalence rate of DME (19) were considered. On the basis of these epidemiological indices, an eligible population of 237,602 individuals was estimated (Table 1). Furthermore, in order to characterize the trend in the number of eligible patients over a 5-year time horizon, an annual rate of population decrease of 0.20% was assumed (18).

**Table 1:** Target population.

**Italian population (2020)**	**59,236,213**
% Annual growth	−0.20%
Type II diabetes prevalence	5.89%
Type II diabetes affected patients	3,489,013
% Patients developing DME	6.81%
DME affected patients	237,602

#### Costs of DME Treatment, Management and Follow-Up

The analysis considered the anti-VEGFs currently authorized in Italy (bevacizumab, ranibizumab and aflibercept) and the sustained-release dexamethasone implant.

To calculate the regimes for the provision of therapeutic alternatives in the Italian setting, a questionnaire was administered to two clinicians (M.V.; F.B.) with proven experience in the management of DME patients. Specifically, the aspects investigated for each treatment analyzed concerned:

- administration frequency on an annual basis;
- number and type of healthcare professionals involved in treatment administration;
- examinations to monitor patients' health condition;
- rate of recourse to interventions following a drug therapy failure (focal laser, vitrectomy);
- percentage of patients undergoing mono- or bi-ocular treatment with a specific drug;
- percentage of the main complications associated with the treatments implying the recourse to either focal laser, vitrectomy or cataract surgery.

The questionnaire allowed us to determine the administration schemes, follow-up and interventions for the management of DME patients, as reported in Table 2.

**Table 2:** Treatment administration schemes, follow-up and interventions for the management of DME patients.

**Treatment with aflibercept**		
**Treatment administration**		
Administration frequency per year		6.00
Duration of administration (minutes)		3.50
**Professionals involved in treatment administration**	***N*** **. healthcare professionals**	**Activity/year (hours)**
Injector physician	1.00	0.35
Nurse	2.00	0.70
Orthoptist	1.00	0.35
Social Health Operator	1.00	0.35
**Follow-up**		**Frequency/year**
Eye examination		5.00
Optical Coherence Tomography (OCT)		5.00
Retinal fluorangiography		1.00
**Interventions following a drug therapy failure**		**Frequency/year**
Focal laser		1.75%
Vitrectomy		1.50%
**Other information**		**%**
% Endophthalmitis (as complication of intravitreal treatment)		0.01%
% Patients on mono-ocular treatment		27.50%
% Patient on bi-ocular treatment		72.50%
**Treatment with ranibizumab**		
**Treatment administration**		
Administration frequency per year		6.00
Duration of administration (minutes)		2.00
**Professionals involved in treatment administration**	***N*** **. healthcare professionals**	**Activity/year (hours)**
Injector physician	1.00	0.20
Nurse	2.00	0.40
Orthoptist	1.00	0.20
Social Health Operator	1.00	0.20
**Follow-up**		**Frequency/year**
Eye examination		5.50
OCT		5.50
Retinal fluorangiography		1.00
**Interventions following a drug therapy failure**		**%**
Focal laser		1.00%
Vitrectomy		1.50%
**Other information**		**%**
% Endophthalmitis (as complication of intravitreal treatment)		0.01%
% Patients on mono-ocular treatment		27.50%
% Patient on bi-ocular treatment		72.50%
**Treatment with bevacizumab**		
**Treatment administration**		
Administration frequency per year		6.00
Duration of administration (minutes)		3.50
**Professionals involved in treatment administration**	***N*** **. healthcare professionals**	**Activity/year (hours)**
Injector physician	1.00	0.35
Nurse	1.50	0.53
Orthoptist	1.00	0.35
Social Health Operator	1.00	0.35
**Follow-up**		**Frequency/year**
Eye examination		7.75
OCT		7.75
Retinal fluorangiography		1.00
**Interventions following a drug therapy failure**		**Frequency/year**
Focal laser		1.00
Vitrectomy		1.00
**Other information**		**%**
% Endophthalmitis (as complication of intravitreal treatment)		0.01%
% Patients on mono-ocular treatment		27.50%
% Patient on bi-ocular treatment		72.50**%**
**Treatment with sustained-release dexamethasone implant**		
**Treatment administration**		
Administration frequency per year		2.50
Duration of administration (minutes)		3.00
**Professionals involved in treatment administration**	***N*** **. healthcare professionals**	**Activity/year (hours)**
Injector physician	1.00	0.13
Nurse	1.83	0.23
Orthoptist	1.00	0.13
Social Health Operator	1.00	0.13
**Follow-up**		**Frequency/year**
Eye examination		3.00
OCT		3.00
Retinal fluorangiography		1.00
**Interventions following a drug therapy failure**		**%**
Focal laser		1.00%
Vitrectomy		1.50%
**Other information**		**%**
Cataract^*^		70.75%
% Endophthalmitis (as complication of intravitreal treatment)		0.01%
% Patients on mono-ocular treatment		27.50%
% Patient on bi-ocular treatment		72.50%

* *Specific complication related to treatment with intravitreal dexamethasone implant, inserted on the recommendation of experts*.

In order to determine the distribution of patients among the pharmacological alternatives considered in the two scenarios under analysis, reference was made to the recourse rates identified in the survey. To define the two scenarios, higher recourse rates were assumed for the dexamethasone implant, and a proportional decrease in the percentages of recourse to anti-VEGFs was assumed. The market share of each treatment and the distribution of patients among the pharmacological alternatives analyzed are reported in the supplementary material (Supplementary Table I).

To determine the direct costs associated with the healthcare professionals involved in providing the alternatives analyzed, we used the annual report of the Agency for Negotiated Representation in Public Administrations, which reports the average per capita salaries in the public administration and in the private sector (20). Furthermore, in order to economically enhance the procedures necessary for the monitoring of DME patients, the Tariff of Outpatient Specialist Services (21) and the National Tariff of Acute Care Services (22) were used. From these references, we obtained the cost per minute of each professional involved and the tariffs associated with each procedure (Supplementary Table II). The formula used to calculate the hourly cost of healthcare professionals is shown below:


$$\begin{array}{l} \text{Hourly}\;\text{cost}=\frac{\text{average}\;\text{gross}\;\text{earnings}}{{\left({\text{n}}^{{}^{\circ}}\;\text{working}\;\text{weeks}\right)}^{*}\left(n^{{}^{\circ}}\;\text{working}\;\text{hours}\;\text{per}\;\text{week}\right)} \end{array}$$

To determine the costs of pharmacological therapies, the Class H Drug Lists (23) was used. In order to estimate the daily cost of purchasing each drug therapy considered, the maximum NHS purchase price of each treatment was considered, from which the cost/mg, weighted by the average daily dose, was calculated (Supplementary Table III), as shown in the following formula:


$$\begin{array}{l} \text{Cost}/\text{mg}=\frac{\text{drug}\;\text{ex}\;\text{factory}\;\text{price}}{\text{mg}\;\text{per}\;\text{package}} \end{array}$$

The mean dosage of each treatment was determined from the results of the survey conducted. Specifically, regarding the drug bevacizumab, and considering the availability of a biosimilar alternative, it was assumed that the original version was used in 50% of cases.

### Indirect Costs

The analysis also took into consideration the productivity loss incurred by patients and their caregivers as a result of the therapy delivered. It was assumed that patients/caregivers were distributed, in terms of employment type, according to the data available in the *Job Pricing: All about Rewards—Salary Outlook 2022 report* (24), which indicates the percentage of workers belonging to four macro-classes of employment (managers, intermediate level managers, office workers, workers/apprentices). The average hourly productivity loss of caregivers, by wage macro-class, was considered (Table 3).

**Table 3:** Hourly earnings by occupational class and percentage of workers/caregivers in each classes.

**Occupational class**	**Annual earnings**	**Hourly earnings**	**% Per occupational class**	
Senior executives	€ 101,096.00	€ 48.60	1.30%	
Managers (intermediate level)	€ 54,136.00	€ 26.03	4.40%	
Office workers	€ 30,770.00	€ 14.79	36.00%	
Workers/Apprentices	€ 24,780.00	€ 11.91	58.30%	
Average hourly loss of productivity			€ 14.05	
**Patient**				
**DME Treatment**	**Hours lost/administration**	**Total hours lost**	**Total costs**	
Aflibercept	6.50	39.00	€ 547.88	
Ranibizumab	6.50	39.00	€ 547.88	
Bevacizumab	6.50	39.00	€ 547.88	
IDI^*^	6.50	16.25	€ 228.28	
**Caregiver**				
**DME Treatment**	**Hours lost/administration**	**Total hours lost**	**% Patients with caregiver**	**Total costs**
Aflibercept	6.50	39.00	65.00%	€ 356.12
Ranibizumab	6.50	39.00	65.00%	€ 356.12
Bevacizumab	6.50	39.00	65.00%	€ 356.12
IDI^*^	6.50	16.25	65.00%	€ 148.38

* *IDI, Intravitreal Dexamethasone Implant*.

To determine the indirect costs incurred by patients/caregivers, the average number of hours devoted to providing treatment and the percentage of patients informally supported by a caregiver were taken from our survey data. The formula for calculating the loss of productivity is the following:


$$\begin{array}{l} \text{Productivity}\;\text{loss}=\frac{\text{average}\;\text{gross}\;\text{earnings}}{{\left(n^{{}^{\circ}}\;\text{working}\;\text{weeks}\right)}^{*}({\text{n}}^{{}^{\circ}}\;\text{working}\;\text{hours}\;\text{per}\;\text{week})}\\ {}*\text{hours}\;\text{destined}\;\text{to}\;\text{therapy}\;\text{provision} \end{array}$$

To estimate these parameters, a questionnaire was administered to an Italian referent (F.A.) of the International Agency for the Prevention of Blindness (IAPB).

## Results

Considering the eligible population with DME and the yearly uptake rate of the dexamethasone implant over the 5-years time interval considered in the analysis, we determined the resource absorption associated with the two scenarios. Table 4 shows the resource consumption per macro cost item and year of analysis.

**Table 4:** Resource absorption: “Scenario AS IS”, “Scenario TO BE” and differential analysis.

	**Year 1**	**Year 2**	**Year 3**	**Year 4**	**Year 5**	**Total**
**Scenario “AS IS”**						
Drug acquisition	€ 892,744,683	€ 890,960,588	€ 889,175,096	€ 887,389,604	€ 885,604,112	€ 4,445,874,083
Healthcare professionals	€ 8,757,871	€ 8,790,509	€ 8,822,494	€ 8,853,882	€ 8,884,678	€ 44,109,434
Interventions	€ 130,225,784	€ 129,965,585	€ 129,706,875	€ 129,448,145	€ 129,189,393	€ 648,535,781
Follow-up	€ 181,805,680	€ 181,291,291	€ 180,778,351	€ 180,267,215	€ 179,757,861	€ 903,900,398
Social costs	€ 163,412	€ 163,085	€ 162,759	€ 162,432	€ 162,105	€ 813,793
Indirect costs—patient	€ 169,764,662	€ 169,425,818	€ 169,086,288	€ 168,746,757	€ 168,407,226	€ 845,430,752
Indirect costs—caregiver	€ 110,319,198	€ 110,099,005	€ 109,878,366	€ 109,657,726	€ 109,437,087	€ 549,391,382
Psychological support	€ 47,851,443	€ 47,755,740	€ 47,660,037	€ 47,564,334	€ 47,468,631	€ 238,300,186
Total/year	€ 1,541,632,732	€ 1,538,451,622	€ 1,535,270,266	€ 1,532,090,094	€ 1,528,911,094	€ 7,676,355,808
Total/cumulative	€ 1,541,632,732	€ 3,080,084,354	€ 4,615,354,620	€ 6,147,444,714	€ 7,676,355,808	
**Scenario “TO BE”**						
Drug acquisition	€ 892,744,683	€ 892,058,802	€ 889,683,098	€ 887,326,977	€ 884,989,591	€ 4,446,803,151
Healthcare professionals	€ 8,757,871	€ 8,717,064	€ 8,707,098	€ 8,696,882	€ 8,686,427	€ 43,565,342
Interventions	€ 130,225,784	€ 133,119,474	€ 134,604,550	€ 136,031,485	€ 137,402,794	€ 671,384,086
Follow-up	€ 181,805,680	€ 180,077,103	€ 179,000,676	€ 177,947,994	€ 176,918,031	€ 895,749,483
Social costs	€ 163,412	€ 163,085	€ 162,759	€ 162,432	€ 162,105	€ 813,793
Indirect costs—patient	€ 169,764,662	€ 167,991,818	€ 166,859,324	€ 165,753,242	€ 164,672,430	€ 835,041,475
Indirect costs—caregiver	€ 110,319,198	€ 109,167,140	€ 108,431,204	€ 107,712,432	€ 107,010,082	€ 542,640,055
Psychological support	€ 47,851,443	€ 47,755,740	€ 47,660,037	€ 47,564,334	€ 47,468,631	€ 238,300,186
Total/year	€ 1,54,632,732	€ 1,539,050,226	€ 1,535,108,745	€ 1,531,195,778	€ 1,527,310,090	€ 7,674,297,571
Total/cumulative	€ 1,541,632,732	€ 3,080,682,958	€ 4,615,791,702	€ 6,146,987,480	€ 7,674,297,571	
**Differential Analysis**						
Drug acquisition	€ 0	€ 1,098,214	€ 508,002	-€ 62,627	-€ 614,521	€ 929,068
Healthcare professionals	€ 0	-€ 73,446	-€ 115,396	-€ 156,99,	-€ 198,251	-€ 544,092
Interventions	€ 0	€ 3,153,890	€ 4,897,675	€ 6,583,340	€ 8,213,400	€ 22,848,305
Follow-up	€ 0	-€ 1,214,188	-€ 1,777,675	-€ 2,319,221	-€ 2,839,830	-€ 8,150,914
Social costs	€ 0	€ 0	€ 0	€ 0	€ 0	€ 0
Indirect costs—patient	€ 0	-€ 1,434,000	-€ 2,226,964	-€ 2,993,515	-€ 3,734,797	-€ 10,389,277
Indirect costs—caregiver	€ 0	-€ 931,865	-€ 1,447,162	-€ 1,945,294	-€ 2,427,006	-€ 6,751,327
Psychological support	€ 0	€ 0	€ 0	€ 0	€ 0	€ 0
Total/year	€ 0	€ 598,604	-€ 161,521	-€ 894,317	-€ 1,601,004	-€ 2,058,238
Total/cumulative	€ 0	€ 598,604	€ 437,083	-€ 457,234	-€ 2,058,238	

The main difference between the use of the dexamethasone implant and anti-VEGFs is the different annual number of administrations; anti-VEGF agents are normally administered 6 times per year, while the dexamethasone implant is associated with an average administration frequency of 2.50 per year. The dexamethasone implant is, however, also associated with a 70.75% frequency of cataract, which is indicated by clinicians as a specific complication of this treatment (Table 2).

Regarding indirect costs, the analysis showed that the average productivity loss per hour was €14.05, that 6.50 h were devoted to drug administration for all the alternatives considered, and that 65% of patients required caregiver support. In the case of anti-VEGFs, the annual indirect cost, obtained by weighting the number of hours by the average hourly cost, and the cost associated with the annual frequency of treatment administration, were €547,88 for the patient and €356,12 for the caregiver; in the case of the dexamethasone implant, the corresponding figures were €228,28 and €148,38. The lower number of administrations required by the dexamethasone implant resulted in lower productivity losses (Table 3).

The cost of drug purchase accounted for the highest absorption of resources over the entire period considered, with an impact of €885,604,112 in 5 years. The macro-item that generated the second-largest absorption of resources proved to be the cost of follow-up, which was €181,805,680 in year 1 and totaled €903,900,398 over the 5-years time horizon considered.

Table 3 shows the absorption of resources related to scenario 2. In this case, too, the greatest cost item proved to be that of drug purchase, which was €892,744,683 in the first year, with an a decreasing trend over the time horizon considered and an impact in the fifth year of €884,989,591. Again, the cost of follow-up was the second-largest item in terms of impact on resources, being €181.805,680 and €176,918,031 in years 1 and 5, respectively.

**Figure 1:**
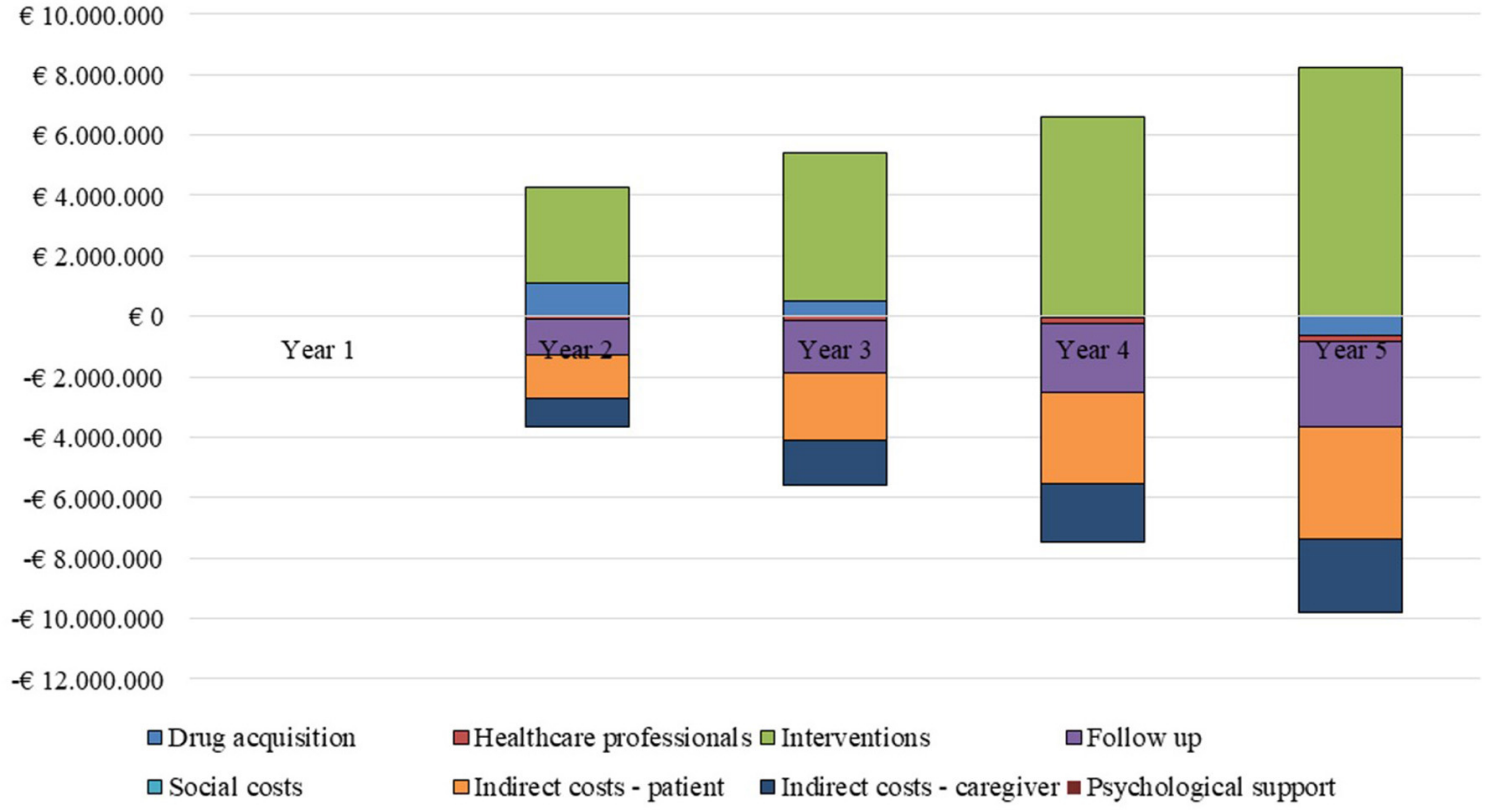
Differential analysis by cost driver and year of analysis.

On comparing the two scenarios (Figure 1), it emerges that the increased use of the dexamethasone implant, assuming a cost of €2,050 per administration, would yield a saving of resources (Table 4).

The higher costs of drug purchase (€929,068) and of the interventions provided (€22,848,305) are offset by the savings obtainable in terms of the reduced workload of healthcare professionals and a decrease in follow-up costs and patients/caregivers' productivity loss. These savings would yield a general saving of €2,058,238 over the 5-years time horizon considered in the analysis.

**Figure 2:**
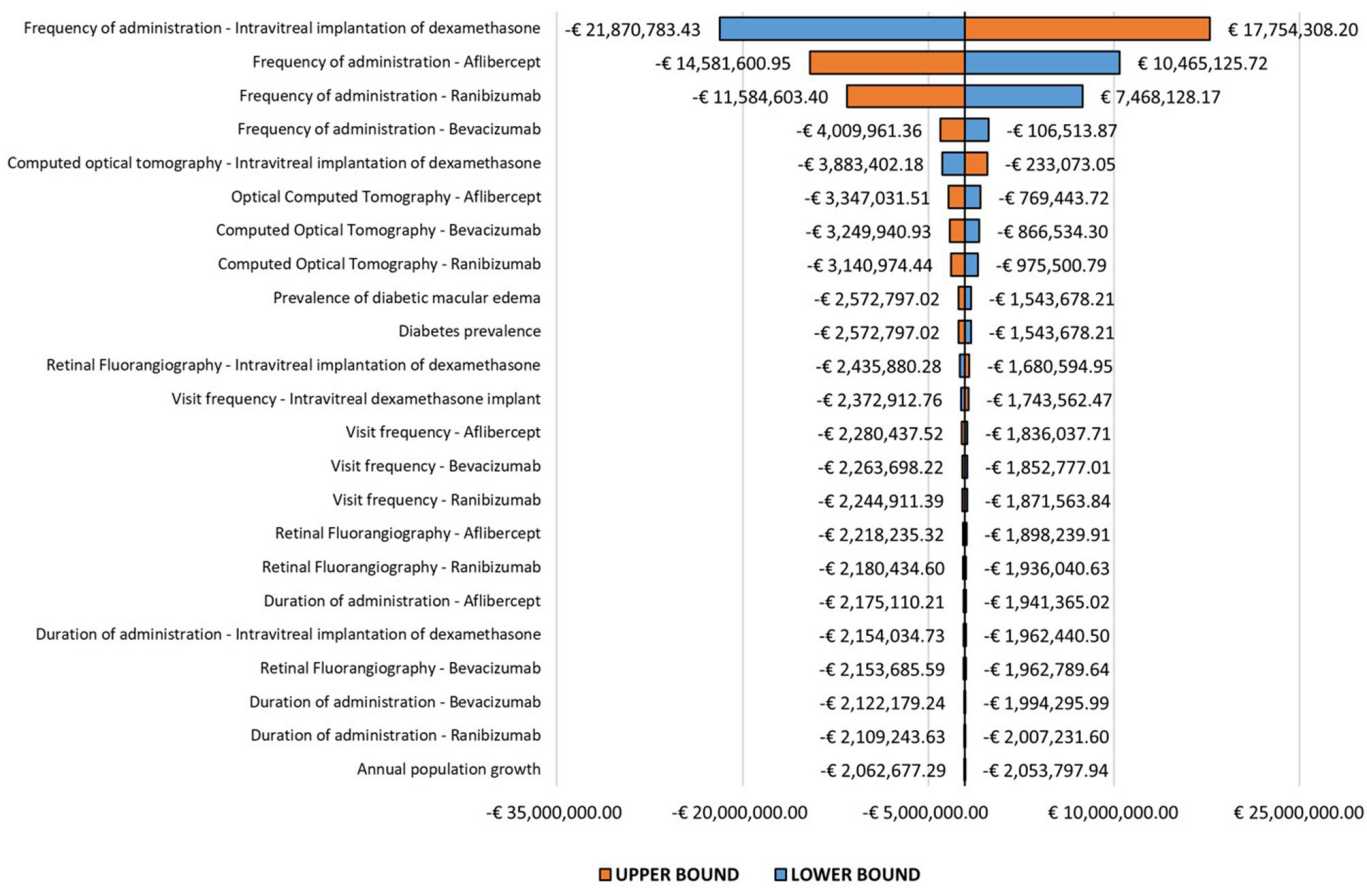
One-way sensitivity analysis.

According to our OWA, the parameter whose variation most significantly influences the base-case result is the administration frequency of the dexamethasone implant (Figure 2) with a trend inversely related to the budget impact results: an increase in the absolute value of this parameter involves, in fact, an increase in expenditure up to €17,754,308.20, while its reduction implies greater savings up to €21,870,783.43.

## Discussion

We estimated the direct and indirect costs of DME management in Italy and analyzed the economic consequences of the increased use of the dexamethasone implant in the Italian setting. Our model considered the NHS perspective and a 5-years time horizon. Furthermore, it also considered the social perspective in terms of lost productivity of patients and their caregivers due to DME management. The input of the economic model was validated by two ophthalmologists and by an Italian referent of the IAPB.

Our analysis involved a comparison between two alternative scenarios for the treatment of DME patients in the Italian healthcare setting: the first scenario was based on current clinical practice (anti-VEGFs as the first-line treatment for DME and the sustained-release dexamethasone implant as the second); the second scenario was based on the assumption of an increased use of the sustained-release dexamethasone implant.

On comparing the two scenarios, it emerged that the increased use of the dexamethasone implant would yield a saving of resources in the management of patients with DME, in that it would reduce the workload of healthcare professionals, follow-up costs, and patients/caregivers' productivity losses. These savings would yield a total saving of € 2,058,238 over the 5-year time horizon considered in the analysis. The main difference between the use of the dexamethasone implant and that of anti-VEGFs lies in the frequency of drug administration; on average, anti-VEGFs are administered 6 times per year, while the dexamethasone implant has an average administration frequency of 2.50 per year. Furthermore, this lower injection frequency results in a lower impact on the productivity losses of patients and their caregivers. Therefore, the use of the dexamethasone implant is an example of DME management that generates not only clinical benefits for patients but also potential savings for the health system and society.

DME is the main cause of vision loss among patients with diabetes worldwide (9) and, with progressive population aging, a greater burden of this disease is expected in the coming years (8). In the last few years, the treatment of this eye disease has been revolutionized by the introduction into clinical practice of intravitreal therapy with anti-VEGF agents and corticosteroid implants (11). Anti-VEGFs, such as ranibizumab, aflibercept and bevacizumab—the latter used as an off-label treatment—have proved much more efficacious than the previous standard of care (laser photocoagulation), achieving clinically relevant improvements in visual acuity (9). Owing to their efficacy and safety profile, anti-VEGFs have become the first-line treatment for DME (15). However, despite their positive impact on the treatment of DME, anti-VEGFs require several intravitreal injections and follow-up visits, resulting in high costs for both the health system (12) and society (13). Furthermore, this need for frequent intravitreal injections is likely to reduce patient compliance and, therefore, clinical efficacy following suspension of the treatment (14).

Corticosteroid implants, such as the sustained-release dexamethasone implant, are usually used as a second-line treatment for patients who do not respond significantly to anti-VEGF injections (15). Increased intraocular pressure (IOP) and cataract are the most common adverse events (AEs) associated with dexamethasone implant treatment. Clinically significant increased IOP are a complication in approximately one third of patients treated with dexamethasone implant. Increases in IOP are most common at 1.5 or 3 months after injection and are typically managed with topical IOP-lowering medication (25). In the MEAD study (26) evaluating dexamethasone implant for the treatment of DME, there were no reports of the development of glaucoma with confirmatory changes in the optic nerve or visual field in the dexamethasone implant 0.7-mg group, and only 1.2% of patients treated with dexamethasone implant compared with 0.3% of patients treated with sham required a laser or surgical procedure for IOP management. Furthermore, Maturi et al. (25) demonstrated that dexamethasone implant has a clear benefit in treating DME despite increases in IOP, and that sequential implants do not have a cumulative effect on IOP.

Despite the potential complications, which must also be considered for other treatments, the use of long-acting drugs can improve the quality of life of patients by reducing the number of intravitreal injections (14). In fact, a reduced number of injections would lead to better management of the visually impaired patient by his caregiver and, therefore, greater compliance with therapy. A greater therapeutic adherence, in turn, would determine a greater efficacy of the treatment and, therefore, a positive impact on visual acuity and the quality of life. Furthermore, these therapies could also lead to a reduction in costs for the health system and society (14).

A recent Italian retrospective study (14) assessed clinical outcomes and the costs of intravitreal drugs. The average cost of intravitreal anti-VEGF therapy was reported to be €261,429 per year, accounting for approximately 16% of the total cost of DME treatment. Ranibizumab had the greatest impact on cost, as it was the most frequently prescribed intravitreal agent (46%), followed by aflibercept (34%). The dropout rate among patients on anti-VEGF agents was 12%. However, when 59% of patients switched to a dexamethasone implant, a significant clinical improvement was achieved. The authors of the study recommended that patients receiving anti-VEGFs with minimal/no clinical benefit should switch to a dexamethasone implant in an attempt to improve vision, lower costs, and reduce the burden of injections on clinics and hospitals (14).

Also according to our analysis, the use of the dexamethasone implant can reduce direct and indirect costs related to DME management in Italy. Furthermore, this treatment modality, owing to the lower frequency of intravitreal injections required, can improve both the quality of life of patients and their compliance with therapy.

Our study has some limitations, the main one being the fact that the time devoted by healthcare professionals to delivering therapies was determined only on the basis of the data collected through our survey of the experts. However, to overcome the lack of robustness associated with this shortcoming and some of the other values considered in the analysis, an OWA was conducted. An element that could significantly influence the results obtained is the percentage of DME patients undergoing bi-ocular treatment, which was estimated as 72.5%, a value that could not be validated by the available scientific evidence. The availability of this datum could significantly improve the estimate made in our analysis.

Furthermore, the insurance costs incurred by patients were not considered in our economic evaluation, as no data were available. Therefore, our calculation of indirect costs may have been underestimated.

Nevertheless, regarding the economic burden of DME in Italy, our study provides new data that can support policymakers in designing value-based strategies to address this growing disease.

Today, in a healthcare context characterized by resource scarcity and increasing service demand, “disinvestment” from low-value services and reinvestment in high-value ones is a key strategy, which can be supported by economic evaluations and health technology assessments (27). Therefore, the availability of therapeutic alternatives that provide both clinical benefits for patients and a reduction in expenditures is crucial, and this also applies to the field of eye diseases such as DME. HTA supports the decision-making processes concerning the use and application of health technologies with scientific evidence (25). Therefore, capacity building of healthcare professionals in this field should also be enhanced in order to implement evidence-based healthcare choices and to ensure proper health governance and value-based application of technological innovations in clinical practice (28).

In conclusion, greater use of the dexamethasone implant could be considered in the Italian healthcare setting in order to improve the vision and quality of life of patients with DME, alleviate the burden of injections, shorten hospital waiting lists and reduce the costs of the health system and society.

## Data Availability Statement

The original contributions presented in the study are included in the article/Supplementary Material, further inquiries can be directed to the corresponding author.

## Author Contributions

Conceptualization and methodology, elaboration of the economic model, and writing—original draft preparation: GC and MB. Definition of the search strategy and writing—review and editing: GC, MB, and RR. Data analysis: MB. Validation of the economic model: MV, FB, and FA. Project administration and funding acquisition: GC. Supervision: GC and AC. All authors have read and agreed to the published version of the manuscript.

## Funding

This study was financed by an unconditional grant from Allergan S.p.A. The sponsor had no role in conducting or designing the study, collecting, analyzing or interpreting the data, or the writing the manuscript. Universitá Cattolica del Sacro Cuore contributed to the funding of this research project and its publication (with funds from UCSC-Line D 2022).

## Conflict of Interest

GC, MB, MV, FA, RR, FB, and AC worked as consultants of HTA Italia Srl, which received funds from Allergan S.p.A. The sponsor had no role in conducting or designing the study.

## Publisher's Note

All claims expressed in this article are solely those of the authors and do not necessarily represent those of their affiliated organizations, or those of the publisher, the editors and the reviewers. Any product that may be evaluated in this article, or claim that may be made by its manufacturer, is not guaranteed or endorsed by the publisher.
