# Supplementary material for: Economic Aspects in the Management of Diabetic Macular Edema in Italy
Source: Front Public Health. 2022 Jul 22;10:938987. doi: 10.3389/fpubh.2022.938987 (PMC9353644; doi:10.3389/fpubh.2022.938987)
Supplement: Supplementary file 1 [file Table_1.DOCX]

Supplementary Material

**Table I.** Recourse rates and patients’ distribution among the therapeutic alternatives

| SCENARIO “AS IS” | | | | | | |
| --- | --- | --- | --- | --- | --- | --- |
|  | | **Year 1** | **Year 2** | **Year 3** | **Year 4** | **Year 5** |
|  | | **Recourse rates** | | | | |
| Aflibercept | | 27.91% | 28.75% | 29.58% | 30.41% | 31.23% |
| Ranibizumab | | 20.93% | 20.09% | 19.25% | 18.43% | 17.60% |
| Bevacizumab | | 9.30% | 9.30% | 9.30% | 9.30% | 9.30% |
| IDI* | | 41.86% | 41.86% | 41.86% | 41.86% | 41.86% |
| TOTAL | | 100.00% | 100.00% | 100.00% | 100.00% | 100.00% |
|  | | **Patients** | | | | |
| Aflibercept | 64,116 | | 65,919 | 67,698 | 69,454 | 71,187 |
| Ranibizumab | 48,087 | | 46,061 | 44,057 | 42,077 | 40,120 |
| Bevacizumab | 21,372 | | 21,329 | 21,287 | 21,244 | 21,201 |
| IDI* | 96,174 | | 95,980 | 95,788 | 95,596 | 95,403 |
| TOTAL | 229,749 | | 229289 | 228,830 | 228,370 | 227,911 |
| SCENARIO “TO BE” | | | | | | |
|  | **Year 1** | | **Year 2** | **Year 3** | **Year 4** | **Year 5** |
| Recourse rates | | | | | | |
| Aflibercept | 27.91% | | 28.38% | 28.90% | 29.41% | 29.90% |
| Ranibizumab | 20.93% | | 19.72% | 18.69% | 17.68% | 16.70% |
| Bevacizumab | 9.30% | | 8.94% | 8.84% | 8.75% | 8.66% |
| IDI* | 41.86% | | 42.96% | 43.57% | 44.16% | 44.73% |
| TOTAL | 100.00% | | 100.00% | 100.00% | 100.00% | 100.00% |
|  | **Patients** | | | | | |
| Aflibercept | 64,116 | | 65,081 | 66,143 | 67,165 | 68,150 |
| Ranibizumab | 48,087 | | 45,222 | 42,759 | 40,374 | 38,065 |
| Bevacizumab | 21,372 | | 20,491 | 20,235 | 19,986 | 19,744 |
| IDI* | 96,174 | | 98,495 | 99,693 | 100,845 | 101,952 |
| TOTAL | 229,749 | | 229,289 | 228,830 | 228,370 | 227,911 |

**IDI: Intravitreal Dexamethasone Implant*

**Table II.** Unit cost of healthcare professionals, examinations, visits and interventions

| HEALTHCARE PROFESSIONALS | | |
| --- | --- | --- |
|  | **Cost/hour** | |
| Injector physician | € 34.76 | |
| Nurse | € 16.08 | |
| Orthoptist | € 16.01 | |
| Social Health Operator | € 14.07 | |
| Psychologist | € 29.65 | |
| EXAMINATIONS, VISITS AND INTERVENTIONS | | |
| Eye examination | | € 12.91 |
| Psychiatric follow-up visit | | € 12.91 |
| OCT | | € 74.88 |
| Retinal fluorangiography | | € 46.48 |
| Focal laser | | € 68.16 |
| Vitrectomy | | € 1,491.00 |
| Treatment of endophthalmitis | | € 1,549.00 |
| Cataract surgery (as complication of intravitreal treatment) | | € 994.00 |

**Table III.** Annual purchase cost per pharmacological treatment

|  | Cost/mg | Recourse rate | Average dose (mg) | Annual frequency | Annual cost |
| --- | --- | --- | --- | --- | --- |
| Aflibercept | € 125.00 |  | 3.600 | 6.00 | € 2,700.00 |
| Ranibizumab | € 195.65 |  | 2.300 | 6.00 | € 2,700.00 |
| Bevacizumab - originator | € 2.00 | 50.00% | 1.250 | 6.00 | € 7.50 |
| Bevacizumab - biosimilar | € 2.04 | 50.00% | 1.250 | 6.00 | € 7.65 |
| IDI* | € 117,142.86 |  | 0.007 | 2.50 | € 2,050.00 |

**IDI: Intravitreal Dexamethasone Implant*

**Table IV.** Hourly earnings by occupational class and distribution of workers/caregivers between working classes

| Occupational class | | Annual earnings | | | Hourly earnings | | | % per occupational class | | |
| --- | --- | --- | --- | --- | --- | --- | --- | --- | --- | --- |
| Senior executives | | € 101,096.00 | | | € 48.60 | | | 1.30% | | |
| Manager (intermediate level) | | € 54,136.00 | | | € 26.03 | | | 4.40% | | |
| Office worker | | € 30,770.00 | | | € 14.79 | | | 36.00% | | |
| Worker / Apprentice | | € 24,780.00 | | | € 11.91 | | | 58.30% | | |
| Average hourly loss of productivity | | | | |  | | | **€ 14.05** | | |
| PATIENT | | | | | | | | | | |
| DME Treatment | | | **Hours lost/administration** | | | **Total hours lost** | | | **Total costs** | |
| Aflibercept | | | 6.50 | | | 39.00 | | | € 547.88 | |
| Ranibizumab | | | 6.50 | | | 39.00 | | | € 547.88 | |
| Bevacizumab | | | 6.50 | | | 39.00 | | | € 547.88 | |
| IDI* | | | 6.50 | | | 16.25 | | | € 228.28 | |
| CAREGIVER | | | | | | | | | | |
| DME Treatment | **Hours lost/administration** | | | **Total hours lost** | | | **% patients with caregiver** | | | **Total costs** |
| Aflibercept | 6.50 | | | 39.00 | | | 65.00% | | | € 356.12 |
| Ranibizumab | 6.50 | | | 39.00 | | | 65.00% | | | € 356.12 |
| Bevacizumab | 6.50 | | | 39.00 | | | 65.00% | | | € 356.12 |
| IDI* | 6.50 | | | 16.25 | | | 65.00% | | | € 148.38 |

**IDI: Intravitreal Dexamethasone Implant*
